# Peripheral Thrombosis and Necrosis after Minimally Invasive Redo Mitral Valve Replacement due to Unknown Etiology: Difficult Diagnosis of Heparin Induced Thrombocytopenia

**DOI:** 10.1155/2015/383104

**Published:** 2015-04-14

**Authors:** Yoshitsugu Nakamura, Daniel T. Bainbridge, Bob Kiaii

**Affiliations:** ^1^Division of Cardiac Surgery, Department of Surgery, London Health Science Centre, University of Western Ontario, 339 Windermere Road, London, ON, Canada N6A 5A5; ^2^Department of Anesthesiology and Perioperative Care, London Health Science Centre, University of Western Ontario, 339 Windermere Road, London, ON, Canada N6A 5A5

## Abstract

We report on a 75-year-old male with acute onset of peripheral thrombosis causing necrosis of the fingers, elbow, and toes associated with thrombocytopenia after minimally invasive redo mitral valve replacement. Both warfarin and dalteparin were commenced on postoperative day 1 and his INR reached 2.1 on postoperative day 4. On postoperative day 5, the patient developed peripheral thrombosis which progressed to necrosis on postoperative day 6. Platelet counts decreased significantly on the same day. His clinical features were compatible with heparin induced thrombocytopenia (HIT). However, serology testing was negative and the diagnosis was never confirmed. The patient was treated for HIT and platelet count improved eventually. Although no clear consensus exists, we believe this case illustrates why therapy for HIT should be initiated when clinical features strongly suggest HIT despite a negative serology test, unless an alternate diagnosis can be found.

## 1. Introduction

In postoperative status of cardiac surgery, the incidence of peripheral thrombosis causing necrosis is extremely rare as long as peripheral circulation is appropriately preserved. Heparin induced thrombocytopenia (HIT) is the most common cause of thrombocytopenia and thrombosis. Therefore, HIT should be the first diagnosis considered when thrombosis or decreased platelet count is seen postoperatively. Serologic testing is usually reliable for the diagnosis of HIT. However, there is no consensus on treatment if serology is negative for patients whose clinical features are compatible with HIT. We report here an example of such a case of a patient chronically on steroids and who presented with peripheral necrosis and thrombocytopenia after minimally invasive mechanical mitral valve replacement.

## 2. Case Report

A 75-year-old male patient with progressive shortness of breath and known history of chronic mitral valve regurgitation following mitral valve repair 19 years ago was admitted to hospital with an episode of dyspnea and syncope. Further investigation revealed severe mitral and tricuspid regurgitation. His past medical history included hypertension, hypercholesterolemia, and chronic atrial fibrillation treated with warfarin. He was also on prednisolone 7.5 mg for follow-up of pituitary macroadenoma resected 15 years ago. He did not have a history of any hypercoagulable disorders including protein S/C deficiency. He underwent a right minithoracotomy redo mechanical mitral valve replacement and tricuspid valve repair.

**Figure 1:**
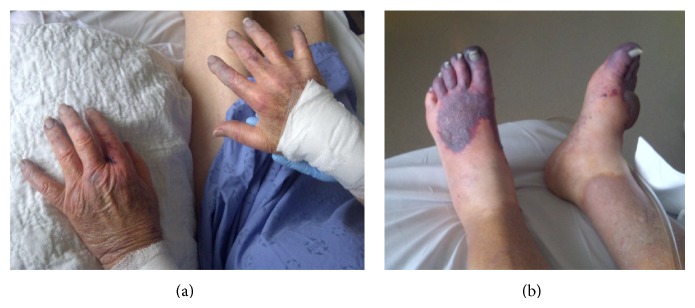
(a) Necrosis and bullous type blisters on the patient's toes. (b) Necrosis in the patient's fingers.

**Figure 2:**
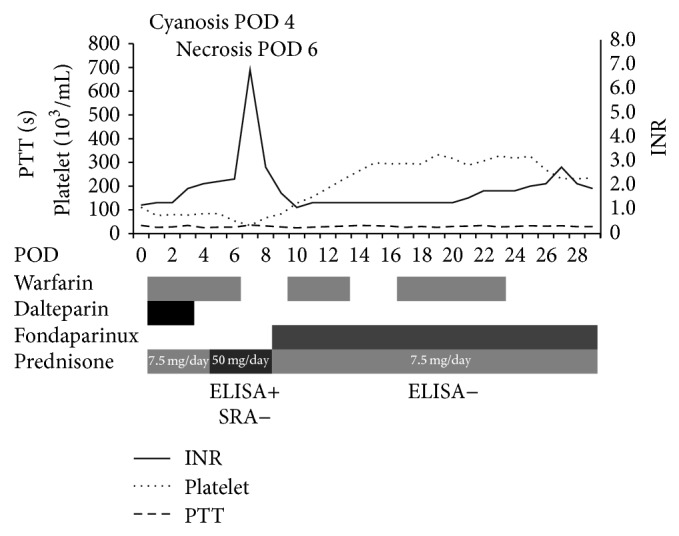
Clinical course. POD: postoperative day, ELISA: enzyme-linked immunosorbent assay, and SRA: serotonin release assay.

His course in the Intensive Care Unit was initially uncomplicated, and he was extubated on postoperative day (POD) 1. Warfarin (5 mg) and dalteparin (17000 units) were commenced on POD 1 and his INR reached 2.1 on POD 4. Prednisolone (7.5 mg) was restarted on POD 1. Starting on POD 4, there was progressive worsening of respiratory parameters, and prednisolone was increased to 50 mg for 4 days. On POD 5, he began to develop toe and finger cyanosis which eventually progressed to necrosis on POD 6 (Figure 1). His preoperative platelet count was 148,000/*μ*L and it decreased to 76,000/*μ*L on POD 1. A further drop occurred on POD 6 and the nadir of the platelet count was 34,000/*μ*L on POD 7 (Figure 2). An enzyme-linked immunosorbent assay (ELISA) for HIT was done and reported as positive, with an optical density equal to 1.09 U (normal < 0.4 U). Therefore he was diagnosed as having HIT. His dalteparin and warfarin were discontinued. He was switched initially to argatroban and then to fondaparinux. In addition, warfarin was restarted on POD 10. There was an improvement overall to the finger and toe cyanosis by POD 10. Bullous type blisters and necrosis developed on his feet and left elbow, for which debridement was performed. All through his postoperative course, the pulse of his bilateral radial and anterior tibial arteries was well palpable. Eventually his platelet count recovered to 300,000/*μ*L by POD 15. However, the serotonin release assay (SRA) which had been sent on POD 6 came back negative on POD 18. Then a repeat ELISA was sent and came back as also negative on POD 18.

His liver and renal function had been stable and within normal range. Immunologic testing showed CH50 elevated at 86, normal C3 and C4 levels, CRP elevated at 140, and negative rheumatoid factor. Anti-nuclear antibody was weakly positive and a mixed pattern.

His respiratory status deteriorated gradually necessitating reintubation on POD 9. He developed increased consolidation of his right lung due to pneumonia, preventing weaning from the ventilator, and died on POD 31.

## 3. Discussion

This patient was initially diagnosed as having HIT due to clinical features compatible with HIT. However, serology testing was negative and the diagnosis was never confirmed.HIT is a complication of heparin administration leading to the most frequent drug-induced, immune-mediated type of thrombocytopenia [1]. HIT after cardiac surgery has been reported to be as high as 1–3% by POD 5 to 10, and 50% of patients with HIT develop venous or arterial thrombotic complications [1, 2]. The diagnosis of HIT is based on both clinical features and serologic testing. The 4 Ts Clinical Scoring System (Thrombocytopenia plus Thrombosis plus Timing in the absence of oTher explanations) is useful to predict the likelihood of HIT [2–4]. The score in this case was 7 of 8 as follows, which indicated a high likelihood of HIT.

*Thrombocytopenia.* The nadir of the platelet count was 34,000/*μ*L (over 50% fall from baseline and platelet nadir > 20,000/*μ*L (score 2)).

*Thrombosis.* This patient had a new thrombosis resulting in skin necrosis after heparin bolus (score 2).

*Timing.* The platelet count dropped twice: the first, on POD 1, and the second, on POD 6. The first platelet drop was likely to be secondary to hemodilution and platelet consumption. The timing of the second drop of the platelet count on POD 6 was compatible with HIT (score 2).

*Other Explanation.* Other differential diagnoses, vasculitis, disseminated intravascular coagulation, low output states, warfarin induced skin necrosis, and preoperative existing peripheral vascular disease, were unlikely, because the patient had no prior history, and, moreover, tests and clinical features for these states were negative or deemed extremely unlikely (score 1).

A difficult point for diagnosis in this case was interpretation of serology tests. Serologic tests for HIT are normally very reliable, especially for ruling out HIT. Thus there is no guideline for the treatment of HIT when serological testing is negative despite the demonstration of clinical features compatible with HIT. Heparin given during cardiopulmonary bypass is remarkably immunogenic, as 25 to 50% of postcardiac surgery patients develop heparin-dependent antibodies during the next 5 to 10 days [5, 6]. Although ELISA tests have high sensitivity (greater than 97%), their specificity (74 to 86%) is limited by the fact that they also detect antibodies in patients who do not have HIT. Therefore, the positive predictive value of the immunoassay can be low, but the negative predictive value is high. Meanwhile, SRA has high sensitivity (88 to 100%) and specificity (89 to 100%) [5]. The probability of false negatives in both the ELISA and SRA is extremely low, and HIT can in general be ruled out when they are negative. Nevertheless, in the current case, there was a questionable result of serology test. The result of the first ELISA on POD 7 was positive. However, the second ELISA tested negative as well as the SRA on POD 18. This interval between the first ELISA and second ELISA was only 11 days, too short a time for negative conversion of HIT antibodies, based on the fact that it takes about 80 days for HIT antibodies to decline to nondetectable levels by ELISA [1]. These conflicting logic-defying results led us to hypothesize that prolonged steroid use in this patient might have had an effect on the serological test results. After all, steroids are well known to suppress immune response generically, so it is possible that steroids directly affect pathological immune responses such as HIT or affect serological antibody-based tests. However, we were unable to uncover any examples of this in the literature, and the role the use of steroids may have had in leading to the negative results for the HIT serologic test remains a conjecture. Supposing that the serological test had been correct in ruling out HIT, nothing else in the differential diagnosis for thrombocytopenia seemed like an obvious alternative candidate for the patient's clinical presentation. There is one possibility in a disease entity called pseudo-HIT. Its clinical features mimic HIT, producing thrombocytopenia 5–14 days after heparin delivery, but serology tests are negative. It is believed to be frequent in adenomas, septicemia, pulmonary embolism, anti-phospholipid antibody syndrome, infectious endocarditis, and diabetic ketoacidosis. Although our patient had none of these, it seems that the course and features were consistent with this clinical entity.

The treatment for HIT is (1) heparin discontinuation, (2) substitution of an alternative anticoagulant such as argatroban or fondaparinux, (3) discontinuation of warfarin, because it can increase the risk of bleeding and result in possible warfarin induced hypercoagulopathy, and (4) adjunctive therapy such as thrombectomy or debridement of necrotic tissue [1]. In the current case, the above therapy was initiated on POD 6 when clinical features strongly suggested HIT. Although we were never able to prove HIT and the final diagnosis remains unknown, we never restarted heparin and continued to treat the patient as though he had HIT. As a result, the thrombocytopenia recovered and necrosis was confined to a small area. In a case such as ours in which clinical features point to HIT but could not be confirmed, no guidelines exist for treatment. However, treatment of HIT is not complicated, and the risks of employing alternative anticoagulation are few. Therefore, we believe that, in such situations, unless an alternative diagnosis is reached, patients should be treated for HIT. The one caveat is that, in pseudo-HIT, heparin may be useful. Lastly, we note that our patient died from refractory respiratory failure but believe that the respiratory failure was unrelated to the complications of HIT.

In summary, we experienced a case of peripheral thrombosis and thrombocytopenia after minimally invasive redo mitral valve replacement. Although we were unable to confirm our diagnosis of HIT because of negative serology, we treated it as such based on the clinical features observed, and the thrombosis and thrombocytopenia improved. In cases such as ours, we believe HIT treatment should be initiated and continued unless other diagnoses are found.
